# A Comparison Study of Age and Colorectal Cancer-Related Gut Bacteria

**DOI:** 10.3389/fcimb.2021.606490

**Published:** 2021-04-30

**Authors:** Yu-Kun Zhang, Qian Zhang, Yu-Liuming Wang, Wei-Yuan Zhang, Han-Qing Hu, Hong-Yu Wu, Xiang-Zong Sheng, Kang-Jia Luo, Hao Zhang, Meng Wang, Rui Huang, Gui-Yu Wang

**Affiliations:** ^1^ Department of Colorectal Cancer, The 2nd Affiliated Hospital of Harbin Medical University, Harbin, China; ^2^ Department of Colorectal Cancer, Cancer Hospital of the University of Chinese Academy of Sciences (Zhejiang Cancer Hospital), Hangzhou, China

**Keywords:** gut microbiota, colorectal cancer, Illumina Miseq sequencing, 16S rRNA, cancer biomarker

## Abstract

Intestinal microbiota is gaining increasing interest from researchers, and a series of studies proved that gut bacteria plays a significant role in various malignancies, especially in colorectal cancer (CRC). In this study, a cohort of 34 CRC patients (average age=65 years old), 26 young volunteers (below 30 years old), and 26 old volunteers (over 60 years old) was enrolled. 16S ribosomal RNA gene sequencing was used to explore fecal bacteria diversity. The operational taxonomic unit (OTU) clustering analysis and NMDS (non-metric multidimensional scaling) analysis were used to separate different groups. Cluster of ortholog genes (COG) functional annotation and Kyoto encyclopedia of genes and genomes (KEGG) were used to detect enriched pathways among three groups. Community separations were observed among the three groups of this cohort. *Clostridia*, *Actinobacteria*, *Bifidobacterium*, and *Fusobacteria* were the most enriched bacteria in the young group, old group, and CRC group respectively. Also, in the young, old, and CRC group, the ratio of *Firmicutes/Bacteroidetes* was increased sequentially despite no statistical differences. Further, COG showed that transcription, cell wall/membrane/envelope biogenesis, inorganic ion transport and metabolism, and signal transduction mechanisms were differentially expressed among three groups. KEGG pathways associated with ABC transporters, amino sugar and nucleotide sugar metabolism, arginine and proline metabolism, and aminoacyl-tRNA biosynthesis also showed statistical differences among the three groups. These results indicated that the intestinal bacterial community varied as age changed and was related to CRC, and we discussed that specific bacteria enriched in the young and old group may exert a protective function, while bacteria enriched in the CRC group may promote tumorigenesis.

## Introduction

Colorectal cancer (CRC) is the third most commonly diagnosed cancer with approximately 1.4 million new cases and 694,000 deaths per year worldwide (Bray et al., 2018). Moreover, its incidence has been rapidly rising in people under the age of 50 over the last 20 years (Patel and Ahnen, 2018). Significant attention has been given to CRC and opened wider windows for its diagnosis and therapeutic strategies, yet its etiology remains vague.

Recently, studies have implied that the intestinal microbiome cannot be ignored (Song and Chan, 2019). The microbiome in human intestinal tract is regarded as the second genome of human beings and plays an important role in intestinal homeostasis. Approximately 10^4^ bacterial species are colonized in the gut of human (Tjalsma et al., 2012). Among the intestinal flora, probiotics such as *Lactobacillus plantarum* and *Saccharomyces* are beneficial to intestinal metabolism; conversely, human *Papillomaviruses* and *Helicobacter pylori* are commonly recognized as the chief agents in cervical cancer and gastric cancer respectively (Zur Hausen, 2009).

Some novel findings have determined the roles of bacteria in CRC. *Fusobacterium nucleatum* was found to be enriched in both stools and tumor tissues of CRC patients, and promotes the tumorigenesis and chemoresistance of CRC (Luo et al., 2019). *Peptostreptococcus anaerobius* was reported as a promotor in colorectal carcinogenesis by modulating tumor immunity (Long et al., 2019). In the past ten years, related studies have created a new field of tumor research, that is, microorganisms, which can be used as biomarkers in combination with conventional diagnostic methods. Targeted gut bacteria treatment methods were also emerging, including selective elimination of carcinogenic flora, fecal transplantation of beneficial bacteria, taking oral probiotics, and so on. However, due to the multiple effects of the microbiome on host biology, it is necessary to carefully consider whether the above treatments have side effects (Janney et al., 2020).

Studying the different bacteria between healthy volunteers and CRC patients is a common way method for the treatment and mechanism exploration of CRC. For example, seven CRC-enriched bacteria (*Bacteroides fragilis*, *Fusobacterium nucleatum*, *Porphyromonas asaccharolytica*, *Parvimonas micra*, *Prevotella intermedia*, *Alistipes finegoldii*, and *Thermanaerovibrio acidaminovorans*) have been identified as potential diagnosis markers for CRC (Dai et al., 2018). As a genetic marker of *Lachnoclostridium*, m3 showed a high value in the non-invasive diagnosis of colorectal adenoma (Liang et al., 2020). Furthermore, circulating bacterial DNA in serum had the potential for early diagnosis and prediction of CRC (Liang et al., 2020). These studies greatly promoted the non-invasive diagnosis technology of CRC.

However, intestinal flora is different in people with different ages (Odamaki et al., 2016). Previous studies only compared the differences of gut bacteria between healthy volunteers and CRC patients, but in the group of healthy volunteers, the dominant gut bacteria of young and old volunteers may be different and protect people from CRC; conversely, specific bacteria in the gut of CRC patients may promote CRC. In this study, in order to profile the intestinal microbiota among the young and old healthy volunteers and CRC patients, high-throughput DNA sequencing technology was performed, and we found that age should be taken into account when using gut bacteria as biomarkers of CRC.

## Material and Methods

### Sample Collection

The fecal specimens of CRC patients (average age=65 years old) were obtained from the department of CRC surgery, while the samples of old and young volunteers were obtained from the physical examination center in the Second Affiliated Hospital of Harbin Medical University. The entry criteria of this study were (1): Volunteers were confirmed to be healthy with a physical examination in the Second Affiliated Hospital of Harbin Medical University and (2) CRC patients who were confirmed by biopsy and imaging evaluation. The exclusion criteria were as follows: (1) Individuals who took antibiotics, corticosteroids, or probiotics within 3 months before sample collection; (2) Individuals who received abdominal surgery or other invasive treatment within 3 months before sample collection; (3) Individuals who used evacuant or underwent colonoscopy within 1 week before sample collection; (4) Individuals with a history of cancer or inflammatory bowel disease; (5) Individuals with a special diet; (6) Individuals who received fecal microbiota transplantation therapy; and (7) Individuals with incomplete information or without informed consent. The demographic characteristics were shown in **Table 1**. Stool was snap frozen in liquid nitrogen for 30 seconds and stored at -80 °C until DNA extraction.

**Table 1:** Demographic characteristics of the three groups.

Characteristics	Young volunteers	Old volunteers	CRC patients
**Sex (%)**			
Males	19(73%)	14(54%)	28(82%)
Females	7(27%)	12(46%)	6(18%)
**Age (Year)**			
Median	23	66	63
Range	21-26	60-89	28-81
**BMI (kg/m^2^)**			
Median	21.55	23.57	23.11
Range	17.3-25.7	16.53-34.01	16.33-32.87
**TNM Staging (%)**			
I			3(8.85%)
II			15(44.1%)
III			13(38.2)
IV			3(8.85%)
**Pathological classification (%)**			
Adenocarcinoma			30(88.2%)
Mucinous adenocarcinoma			4(11.3%)

BMI, (Body Mass Index).

### DNA Extraction and PCR Amplification

Microbial DNA was extracted from fecal samples using the E.Z.N.A.@ soil DNA Kit (Omega Bio-tek, Norcross, GA, U.S.) according to manufacturer’s protocols. The final DNA concentration and purification were determined by NanoDrop 2000 UV-visspectrophotometer (Thermo Scientific, Wilmington, USA), and DNA quality was checked by 1% agarose gel electrophoresis. The V3-V4 hypervariable regions of the bacterial 16S rRNA gene were amplified with primers:

Forward: 338(5’-ACTCCTACGGGAGGCAGCAG-3’)

Reverse: 806(5’-GGACTACHVGGGTWTCTAAT-3’)

by thermocycler PCR system (GeneAmp 9700, ABI, USA). The PCR reactions were conducted using the following program: 3 minutes of denaturation at 95°C, 27 cycles of 30 seconds at 95°C, 30 seconds for annealing at 55°C, 45 seconds for elongation at 72°C, and a final extension at 72°C for 10 minutes. PCR reactions were performed in triplicate 20 μL mixture containing 4 μL of 5 × FastPfu Buffer, 2 μL of2.5 mM dNTPs, 0.8 μL of each primer (5 μM), 0.4 μL of FastPfu Polymerase, and 10 ng of template DNA. The resulted PCR products were extracted from a 2% agarose gel and further purified using the AxyPrep DNA Gel Extraction Kit (Axygen Biosciences, Union City, CA, USA) and quantified using QuantiFluor ™ -ST (Promega, USA) according to the manufacturer’s protocol.

### Illumina MiSeq Sequencing

Purified amplicons were pooled in equimolar and paired-end sequenced (2 × 300) on an Illumina MiSeq platform (Illumina, San Diego, USA) according to the standard protocols by Majorbio Bio-Pharm Technology Co. Ltd. (Shanghai, China).

### Processing of Sequencing Data

Raw fastq files were quality-filtered by Trimmomatic and merged by FLASH with the following criteria: (i) The reads were truncated at any site with an average quality score <20 over a 50 bp sliding window; (ii) Sequences whose overlap was longer than10 bp were merged according to their overlap with a mismatch no more than 2 bp; and(iii)Sequences of each sample were separated according to barcodes (exactly matching) and primers (allowing 2 nucleotide mismatching), and reads containing ambiguous bases were removed. Operational taxonomic units (OTUs) were clustered with 97% similarity cutoff using UPARSE (version 7.1 http://drive5.com/uparse/) with a novel ‘greedy’ algorithm that performs chimera filtering and OTU clustering simultaneously. The taxonomy of each 16S rRNA gene sequence was analyzed by RDP classifier algorithm (http://rdp.cme.msu.edu/) against the Silva (SSU123) 16S rRNA database using confidence (threshold) of 70%.

### Beta Diversity Analysis

Beta diversity analysis represents a comparison of microbial community composition and is used to assess differences of microbial community composition. The basic output of this comparison is a distance matrix that represents the difference between every two samples in the community. NMDS (non-metric multidimensional scaling) analysis was chosen for the sample similarity comparison among three groups. This is a method of simplifying, analyzing, and categorizing research objects (samples or quantities) in a multi-dimensional space into low-dimensional spaces, while retaining a method for analyzing raw relational data among objects. The basic feature is to regard the similarity or dissimilarity data between objects as a monotonic function of point distance. On the basis of maintaining the original data order relationship, the original data are replaced with new identical data columns for metric multidimensional scaling analysis. This approach simplifies the study objects (samples or quantities) in a multi-dimensional space into low-dimensional spaces for localization, analysis, and categorization, while preserving the original relationships among objects. The basic feature of NMDS is to regard the similarity or dissimilarity data among objects as a monotonic function of point distance and replace the original data with new identical data columns for metric multi-dimensional scaling analysis on the basis of maintaining the original data order.

### Statistical Software

Software mothur (version_1.30.2) was used for Alpha diversity analysis; in Non-metric multidimensional scaling (NMDS) analysis, Qiime (version_1.9.1) was applied to calculate the distance matrix of beta diversity, and then the R package (version_3.4.3) was used for analysis and mapping. LEfSe (http://huttenhower.sph.harvard.edu/galaxy/root?tool_id=lefse_upload) was used for multi-level species difference discriminant analysis; PICRUSt (version_1.1.0) software was used for the function prediction.

### Statistical Analysis

All statistical calculations were performed in R 3.4.3. Kruskal-Wallis H test was used to compare differences among three groups, *P*<0.05 was considered to be statistically significant, and the correction of the *P*-value is responsible for the false discovery rate (FDR).

## Results

### Raw Data Management

86 samples were collected, and 4,920,316 sequence fragments were obtained with a total length of 2,072,157,256 bps. The length of all samples was mainly in the region of 291~451 bp with an average of 422 bp. The raw reads were deposited into the NCBI Sequence Read Archive (SRA, http://www.ncbi.nlm.nih.gov/Traces/sra) database (Accession Number: SRP271491) for the storage and sharing of the sequencing data generated in this study.

### OTU Clustering and Analysis

We further performed OTU clustering on all effective sequences. After the extraction according to the minimum number of sample sequences, we finally obtained 1416 OTU units. For Shannon-wiener, as shown in **Figure S1**, the curve rose rapidly at the beginning, indicating that the number of newly discovered bacteria gradually increased with the sequencing depth. As the sequencing depth increased, the curve became flat and reached a plateau, indicating that the sequencing depth met the requirements and covered almost all strains.

**Figure 1:**
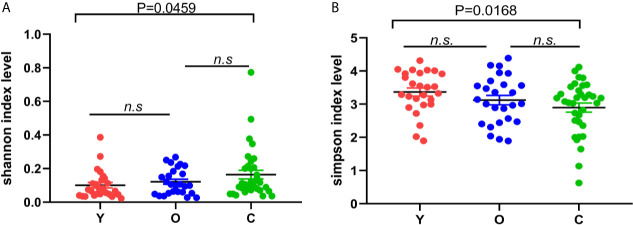
The difference of Shannon **(A)** and Simpson **(B)** index among three groups; the bigger the Shannon index is, the more diverse the sample is, while the Simpson index is the opposite. (Y, young volunteers; O, old volunteers; C, CRC patients). n.s, no significance.

In alpha diversity analysis, there was no significant differences among the three groups, but we found that in the young group, old, and CRC group, Shannon and Simpson indexes were increasing and descending sequentially (**Figures 1A, B**). Between the young and old volunteers, these two indexes showed no statistical differences. But, between the young healthy subjects and CRC group, there was a dramatic difference for both Shannon and Simpson index (*P*=0.0459, 0.0168), indicating that in our study, the community was more diverse in CRC group. Sobs, chao, and ace are indexes that can reflect the community richness of gut bacteria, and we found these three indexes increased sequentially in the young, old, and CRC group despite no statistical differences(**Figure S2**), suggesting that the young volunteers had a greater species abundance.

### Intestinal Flora in Different Groups

**Figure 2:**
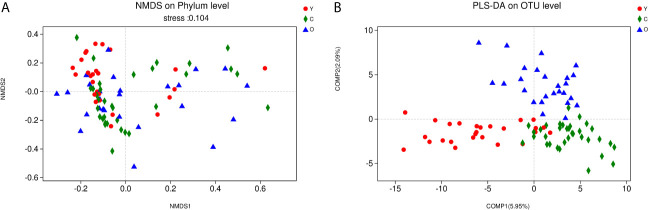
The community difference among the three groups. **(A)** Points of different colors or shapes represent samples of different groups. The closer the two sample points are, the more similar the species composition of the two samples is. Horizontal and vertical coordinates represent relative distances and have no practical significance. The stress number can verify the merits and demerits of Nonmetric multidimensional scaling (NMDS) analysis results. It is generally believed that stress < 0.2 can be expressed by the two-dimensional dot pattern of NMDS, and our grouping scheme has a certain explanatory meaning on the phylum level. **(B)** The scale of X and Y axis is relative distance, which has no practical significance. Comp1 and Comp2 respectively represent the suspected influencing factors that deviate the microbial composition of the three groups of samples. (Y, young volunteers; O, old volunteers; C, CRC patients).

As a dimensionality reduction-based method, NMDS analysis was applied for dissimilarities in the microbial composition among three groups. The results from NMDS analysis on phylum level (**Figure 2A**) was measured by the NMDS intensity index (stress= 0.104 on phylum level). The corresponding value in the other levels were as follows: class (stress=0.136), order (stress=0.138), family (stress=0.206, unexplainable meaning), and genus (stress=0.212, unexplainable meaning). The graphics of other levels (class, order, family, genus) were displayed in (**Figures S3A–D**). Partial least squares-discriminant analysis (PLS-DA) displayed a distinct separation on the OTU level among three groups (**Figure 2B**). These all meant that our grouping method was rational, and the bacterial community differed from each other.

### Dominant Gut Bacterial Community in Multiple Testing

**Figure 3:**
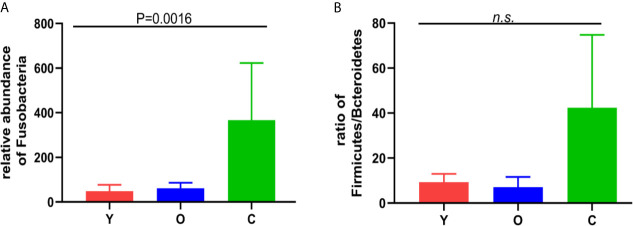
**(A)** the relative abundance of the *Fusobacterium* among the three groups. **(B)** the *Firmicutes/Bacteroidetes* ratio among the three groups. n.s, no significance.

**Figure 4:**
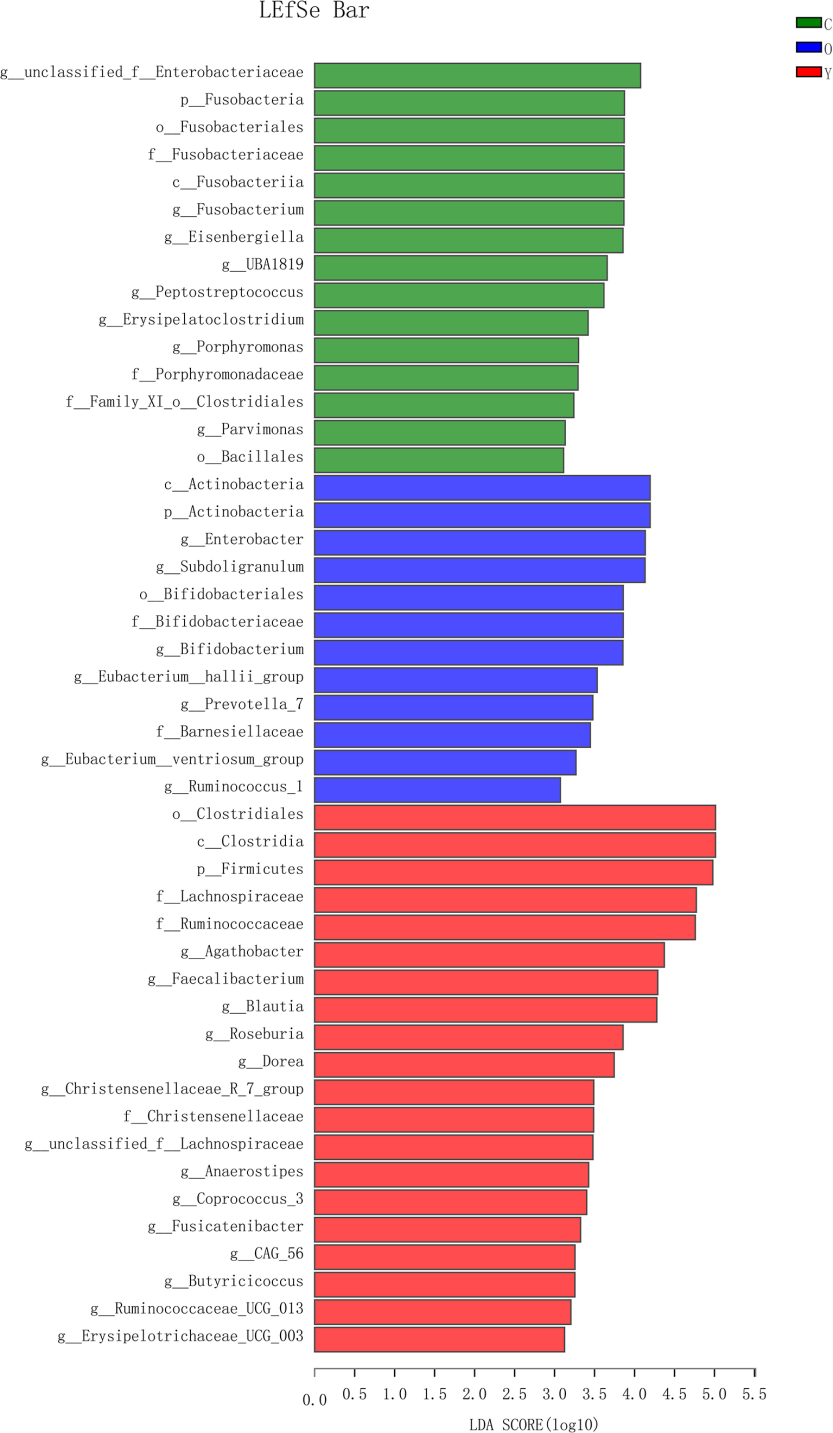
The LDA score obtained by linear regression analysis (LDA), The threshold of linear discriminant analysis score was set as 3, the larger the LDA score is, the greater differences among three groups are. (Y, young volunteers; O, old volunteers; C, CRC patients).

OTU analysis found that *Fusobacteria* showed a significant increasing trend in the young volunteers, old volunteers, and CRC patients sequentially (**Figure 3A**, *P*=0.0016). The *Firmicutes/Bacteroidetes* ratio in CRC group far exceeded the other two groups (**Figure 3B**). The multi-level LEfSe analysis was performed to find the microbial groups with significant effects on multiple groups, and the results of LEfSe among the three groups illustrated that 47 bacterial species abundance had statistically significant differences. Furthermore, 15, 12, and 20 taxa increased in young volunteers, old volunteers, and CRC patients respectively (**Figure 4**). As was shown in **Figure 4**, at the phylum (LDA (linear discriminant analysis) score=3.8716, *P*=0.00189), class (LDA score=3.8701, *P*=0.00189), order (LDA score=3.8725, *P*=0.00189), family (LDA score=3.8691, *P*=0.00326), and genus (LDA score=3.8678, *P*=0.00326) level, increased *Fusobacteria* was detected as the most powerful marker in CRC patients. *Peptostreptococcus* also increased in CRC patients on genus level (LDA score=3.6172, *P*<0.0001). Besides, *Actinobacteria* in the phylum (LDA score=4.1951, *P*=0.04396) and class (LDA score=4.1951, *P*=0.04396) level, and *Bifidobacterium* in order (LDA score=3.8589, *P*=0.02069), family (LDA score=3.8589, *P*=0.02069), and genus (LDA score=3.8552, *P*=0.02147) level showed a greater abundance in old volunteers. Two butyrate -producing species, *Clostridia* in class (LDA score=5.0127, *P*=0.00151) and order (LDA score=5.0128, *P*=0.0015) level and *Firmicutes* in phylum (LDA score=4.9796, *P*=0.003) level, were abundant in young volunteers (**Figure 4**). Again, a multi-level kruskal-wallis test was performed to confirm the findings of LEfSe analysis, and the significant differences of specific bacteria among the three groups could be observed (**Table 2**, **Figures S4A–E**). As was shown in **Table 2**, *Clostridia* was enriched in the young group on class (*P*=0.00151) and order (*P*=0.001497) level. In the old group, *Actinobacteria* was more abundant on phylum and class level (*P*=0.04445), and *Bifidobacteriales* was more abundant on order (*P*=0.02087), family (*P*=0.02087), and genus (*P=*0.02175) level; *Fusobacteria* was more abundant in the level of phylum (*P*=0.001633), class (*P*=0.001633), and order (*P*=0.001633). So, combining the results of two tests comprehensively, the specific bacteria in gut bacterial composition of the young, old, and CRC group were *Clostridia*, *Actinobacteria* and *Bifidobacteriales*, and *Fusobacteria*, respectively.

**Table 2:** Taxa differentially represented in the gut microbiomes of the three groups.

Taxa	Young group (%)	Old group (%)	CRC group (%)	*P*-value
**Phylum**				
*Firmicutes*	61.56 ± 18.99	45.53 ± 19.03	48.85 ± 18.53	0.002988
*Actinobacteria*	3.75 ± 4.667	5.172 ± 7.796	2.015 ± 2.754	0.04445
*Fusobacteria*	0.2109 ± 0.6064	0.2629 ± 0.5455	1.57 ± 6.375	0.001633
**Class**				
*Clostridia*	50.42 ± 20.5	34.78 ± 21.22	31.15 ± 19.33	0.00151
*Actinobacteria*	3.75 ± 4.667	5.172 ± 7.796	2.015 ± 2.754	0.04445
*Fusobacteria*	0.2109 ± 0.6064	0.2629 ± 0.5455	1.57 ± 6.375	0.001633
*Alphaproteobacteriales*	0.01481 ± 0.06517	0.001152 ± 0.005069	0.0273 ± 0.1156	0.03095
*unclassified_p_Firmicutes*	0.02418 ± 0.04222	0.002303 ± 0.007181	0.006793 ± 0.01038	<0.001
**Order**				
*Clostridiales*	50.42 ± 20.5	34.78 ± 21.22	31.14 ± 19.33	0.001497
*Bifidobacteriales*	2.209 ± 3.584	2.837 ± 5.602	1.225 ± 2.464	0.02087
*Fusobacteriales*	0.2109 ± 0.6064	0.2629 ± 0.5455	1.57 ± 6.375	0.001633
*Bacillales*	0.005593 ± 0.0067	0.04146 ± 0.08009	0.2883 ± 0.8524	0.004154
**Family**				
*Lachnopiraceae*	27.87 ± 15.53	16.41 ± 11.44	16.06 ± 12.34	0.003683
*Ruminococcaceae*	20.72 ± 14.26	15.45 ± 14.24	10.28 ± 9.682	0.005064
*Bifidobacteriaceae*	2.209 ± 3.584	2.837 ± 5.602	1.225 ± 2.464	0.02087
**Genus**				
*Faecalibacterium*	7.981 ± 7.474	4.987 ± 7.052	3.996 ± 5.404	0.023
*Bifidobacterium*	2.208 ± 3.583	2.82 ± 5.592	1.223 ± 2.463	0.02175
*Blautia*	6.448 ± 8.051	3.277 ± 3.678	2.895 ± 5.211	<0.001
*Agathobacter*	5.883 ± 7.674	1.873 ± 2.434	1.258 ± 2.217	0.003082

Significant differences among three groups based on the acquired species abundance data by Kruskal-Wallis test.

### Functional Differences Among the Three Groups

**Figure 5:**
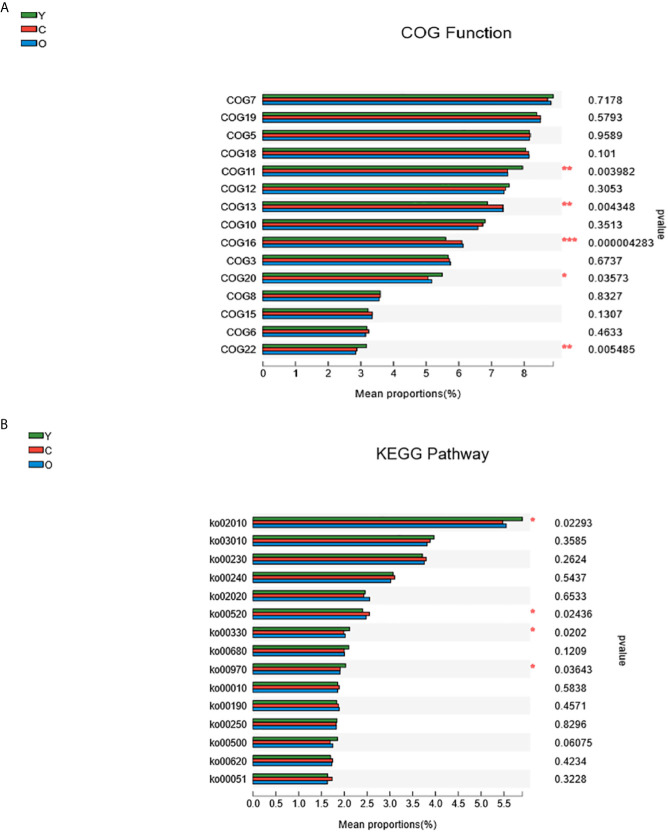
The function prediction of the three groups. **(A)** The differences of Cluster of Ortholog Genes (COG) function. **(B)** The abundance differences of the Kyoto Encyclopedia of Genes and Genomes (KEGG) pathway. (0.01 < *P* ≤ 0.05 marked as *, 0.001 < *P* ≤ 0.01 marked as **P ≤ 0.001 marked as***). (Y, young volunteers; O, old volunteers; C, CRC patients).

Using PICRUST software, we implemented the functional prediction of the 16S rRNA, and the results of Cluster of ortholog genes (COG) functional annotation showed that these functions were different from each other in transcription (COG11, P=0.003982), cell wall/membrane/envelope biogenesis (COG13, P=0.004348), inorganic ion transport and metabolism (COG16, P=0.000004), signal transduction mechanisms (COG20, P=0.03573), and defense mechanisms (COG22, P=0.005485) (**Figure 5A**). According to the Kyoto encyclopedia of genes and genomes (KEGG) database, we found four significant pathways enriched among three groups. They were associated with ABC transporters (KEGG02010, P=0.02293), amino sugar and nucleotide sugar metabolism (KEGG00520, P=0.02436), arginine and proline metabolism (KEGG00330, P=0.0202), and aminoacyl-tRNA biosynthesis (KEGG00970, P=0.03643) (**Figure 5B**).

## Discussion

In this cohort study, although there were no statistical differences in alpha diversity among the three groups, the results indicated that the CRC group had higher community diversity (**Figures 1A, B**). We also found that the CRC group had a lower abundance according to community richness index (**Figure S2**). We also found several bacterial species contributing to the community disparity among the three groups (**Figures S4A–E**; **Table 2**). *Clostridia, Fusobacteria, Actinobacteria*, and *Bifidobacterium* could be potential biomarkers for the diagnosis of CRC in people with different ages. In addition, we observed different microbiome function abundances by 16S rRNA function prediction among three groups. These results are consistent with the former studies which focused on the correlation of the gut microbiota and CRC (Ahn et al., 2013; Liang et al., 2019), but there also existed reverse findings compared with the previous study, such as that the community diversity was higher in the CRC group (Yang et al., 2019). We thought two factors would cause this consequence: the diet of people in this study is different from previous studies and the sample size in this study was smaller.

The gut microbiome can regulate biological homeostasis through the metabolism, human immune system, obesity, diabetes, and various types of cancer (Gagliani et al., 2014; Komaroff, 2017; Al Nabhani and Eberl, 2020). Simultaneously, age, diet, and tumor can affect the bacterial community (Mariat et al., 2009; Arkan, 2017; Deshpande et al., 2018). This bidirectional relationship requests more accurate research designates. So, in this study, in order to explore the bacterial composition affected by age and tumor, we enrolled three groups of young volunteers, old volunteers, and CRC patients.

In the young group, *Firmicutes* in phylum and class level, *Clostridia* in class and order level, and *Blautia* in genus level were significantly higher. These three species are the main producers of butyrate in the intestinal tract. Butyrate is an important energy source in the colonic epithelial cell, and could also regulate the proliferation and differentiation of colonic epithelial cells. Meanwhile, through inhibiting the release of TNF-α, NO, and other inflammatory mediators, and up-regulating P53 gene expression (Vipperla and O’Keefe, 2012; Anantharaju et al., 2016), butyrate exerted an anti-cancer function. In addition, *Blautia*, as an acetate-producer, can suppress inflammation and cancer in colon and rectum (Louis et al., 2014; Tong et al., 2018; Laffin et al., 2019). The abundance of *Faecalibacterium* in the genus level is about 2-fold of the CRC group. In accordance with the present study (Yang et al., 2019), cancer patients with abundant *Faecalibacterium* colonizing in their gut would acquire a better response and prognosis after chemotherapy (Routy et al., 2018; Gopalakrishnan et al., 2018). To sum up, these commensal species colonizing in the gut lumen of young people may play a beneficial role in avoiding inflammation and tumorigenesis.

At the level of phylum and class, *Actinobacteria* could be a biomarker in the old group in this study. *Actinobacteria* is a large family of human gut microbiome (Kundu et al., 2017), and recently, a study isolated a secondary metabolite of a specific *Actinobacteria* sp. from a healthy volunteer (Zhou et al., 2017). One of its secondary metabolites, actinomycetes, has a strong suppression function in multiple tumor cell lines. Ravikumar et al. also found anticancer impact of sediment actinomycetes on MCF-7 and MDA-MB-231 cell lines (Ravikumar et al., 2012; Rangan and Hang, 2017). So, we speculated that specific *Actinobacteria* in the old volunteers may produce a protective effect. The richer *Bifidobacteriaceae* was colonized in the intestine of the old volunteers in the level of order, family, and genus. It has been reported to maintain the intestinal immunity balance, and mitigate intestinal immunopathology in the context of CTLA-4 blockade (Ruiz et al., 2017; Song and Chan, 2019). Thomas F et al. found commensal *Bifidobacterium* can promote antitumor immunity and facilitate anti-PD-L1 efficacy (Wong and Yu, 2019). What interested us was that the abundance *of Akkermansia* in the old group was more than twice as many as the other two groups in the family and genus level (**Figure S4**). Oral administration of specific *Bifidobacterium* strains had been reported to increase the abundance of *Akkermansia* and inhibit intestinal inflammation. A recent study proved that Amuc_1100 of *Akkermansia* restrained colitis-associated CRC (Everard et al., 2013; Cani, 2019; Wang et al., 2020). This indicated the *Bifidobacterium* may play protective roles by changing the abundance of other beneficial bacteria.

A previous study revealed that gender and age had the strongest association with the composition of gut bacteria (Byrd et al., 2021). And in our study, after analyzing the different dominant bacteria of the two healthy groups, we can conclude that in the young and old volunteers, specific gut microbiomes can help to maintain their intestinal homeostasis.

*Fusobacteria* showed an obvious higher concentration in the CRC patients in our study; its abundance in CRC patients is approximately 7.5-fold and 6-fold of the young and old volunteers, respectively. As a common oncogenic bacteria, *Fusobacteria* family combined with CRC cells and immune cells through the adhesion of FadA and Fap2 can affect multiple stages of CRC progression, such as cancer cell proliferation, tumor immune, recurrence, and chemotherapy resistance, and its abundance is remarked as an index to indicate a poor prognosis of CRC patients (Yu et al., 2017; Yamaoka et al., 2018; Brennan and Garrett, 2019; Garrett, 2019; Hashemi Goradel et al., 2019). A higher *Firmicutes/Bacteroidetes* ratio was proven to be a relevant biomarker of gut dysbiosis (Magne et al., 2020), and the dysbiosis of intestinal bacteria was proven as a risk factor of CRC (Janney et al., 2020). In our study, this ratio presented an increasing tendency in the young volunteers, old volunteers, and CRC patients sequentially, despite no significant statistical differences. Besides, the former studies reported that *Bacteroides* predicted a poor response to chemotherapy and poor prognosis in patients (Gopalakrishnan et al., 2018; Routy et al., 2018). The higher abundance of *Bacteroidetes* in CRC patients may also contribute to the carcinogenesis. Taken together, we speculated that the luck of some dominant species in the young and old volunteers, together with the increased *Fusobacteria* abundance, would promote colorectal tumorigenesis.

Through the function prediction, we found that most COG functions and pathways decreased in both the old and CRC volunteers. The young group are featured with richer signal transduction and defense mechanisms, which may develop a protective impact, but there are some exceptions: the function about membrane biogenesis and inorganic ion transport and metabolism and amino sugar and nucleotide sugar metabolism pathways are more abundant in the CRC group (**Figures 5A, B**). Numerous studies have proven that the host and gut microbiome shape each other, and the imbalance of the gut microenvironment is deteriorated during the morbid state, which damages its beneficial effects immensely (Kelly et al., 2015; Ringel, 2017). The function change in the different groups of participants must develop a tumorigenesis or protective effects, and the function mechanism may help scientists to study the malignant occurrence mechanisms and act as a therapeutic target.

Despite the novel findings of this study, there were still some limitations. So, we summarized the strengths and limitations of this study. The strengths of this study were (1): Previous studies have not compared the gut bacteria among young volunteers, old volunteers, and CRC patients; (2) The use of next generation sequencing in this study; (3) The results of NMDS and PLS-DA demonstrated that bacterial community differed among three groups; and (4) We uncovered that there were various dominant bacteria among young volunteers, old volunteers, and CRC patients, and the function prediction was also different among three groups. The limitations of this studies are as follows: (1) The sample size was not too large; (2) The results will be more credible when a group of young CRC patients is added; and (3) In future studies, the Metagenomics Sequencing should be implemented to determine the function prediction of this study.

In conclusion, our study illustrated that the intestine of the young volunteers, old volunteers, and CRC patients were colonized with different microorganisms and we suppose that the same bacteria could exert different functions in different populations, indicating that age should be considered as an important confounder when enrolling cases in population studies. In addition, other microorganisms in the intestine, such as parasites or viruses, showed obvious correlation with CRC. So, to promote the quality of these studies, there is still a long way to go. We hope that this cohort study may offer some guidance for using gut microbes as biomarkers to predict the risk of colorectal cancer, and yield some inspiration to study the oncobacterium and protective bacteria in people with different ages.

## Data Availability Statement

The datasets presented in this study can be found in online repositories. The names of the repository/repositories and accession number(s) can be found below: NCBI, PRJNA64570.

## Ethics Statement

The studies involving human participants were reviewed and approved by Ethics Review committee in the second affiliated hospital of Harbin Medical University. The patients/participants provided their written informed consent to participate in this study.

## Author Contributions

Y-KZ, QZ, Y-LW, and W-YZ are responsible for sequencing and article writing. H-QH, H-YW, X-ZS, K-JL, HZ, and MW are responsible for the collection and processing of clinical samples. RH and G-YW are responsible for the experimental design. All authors contributed to the article and approved the submitted version.

## Funding

This work was supported by the [Applied Technology Research and Development Project of Heilongjiang Province] under Grant [number GA19C003]; [National Natural Science Foundation of China #2] under Grant [number 81872034]; [National Natural Science Foundation of China#3] under Grant[number 81702867]; and the [China Postdoctoral Science Foundation#4] under Grant [number 2019M651319].

## Conflict of Interest

The authors declare that the research was conducted in the absence of any commercial or financial relationships that could be construed as a potential conflict of interest.

## Supplementary Material

Supplementary Figure 1 The Shannon-Wiener curve.

Supplementary Figure 2 The comparison of the sobs、ace, and chao index level among three groups. (Y: young volunteers; O: old volunteers; C: CRC patients).

Supplementary Figure 3
**(A–D)** The results of NMDS on the level of Class, Order, Family, and Genus. (Y: young volunteers; O: old volunteers; C: CRC patients).

Supplementary Figure 4
**(A–E)** Distinct distribution of gut bacterial taxa among the three groups on the level of Phylum, Class, Order, Family, and Genus. (Y: young volunteers; O:old volunteers; C: the CRC patients).
