# Supplementary figures and images for: A Comparison Study of Age and Colorectal Cancer-Related Gut Bacteria
Source: Front Cell Infect Microbiol. 2021 Apr 30;11:606490. doi: 10.3389/fcimb.2021.606490 (PMC8121496; doi:10.3389/fcimb.2021.606490)

Shannon curves

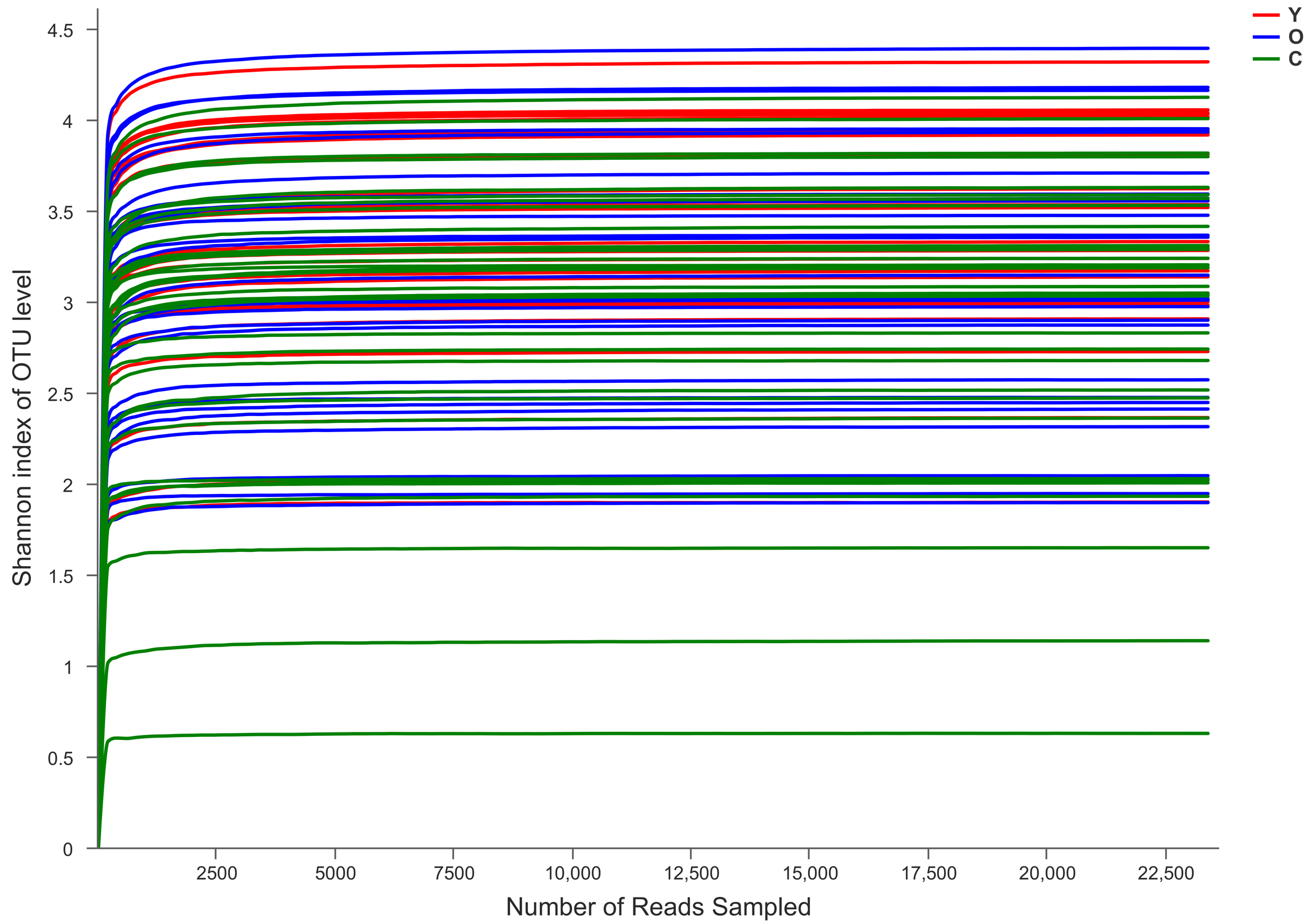

Supplement: Supplementary Figure 1 — The Shannon-Wiener curve. [file DataSheet_1.pdf]

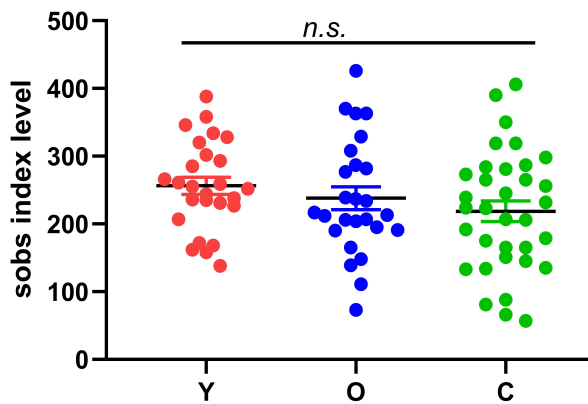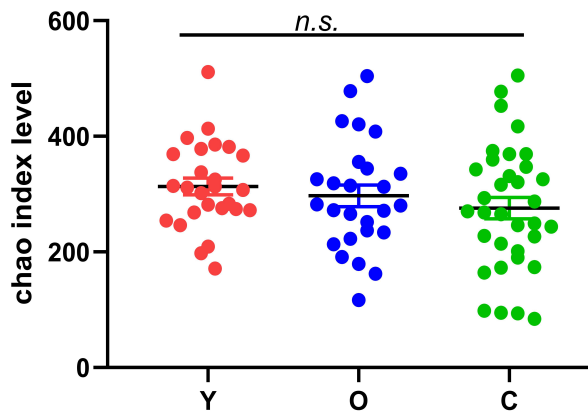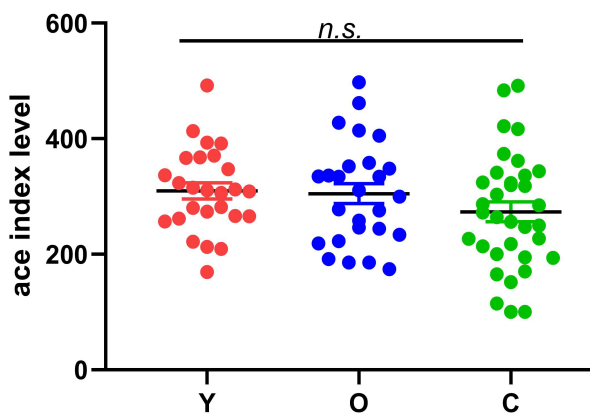

Supplement: Supplementary Figure 2 — The comparison of the sobs、ace, and chao index level among three groups. (Y: young volunteers; O: old volunteers; C: CRC patients). [file DataSheet_2.pdf]

A

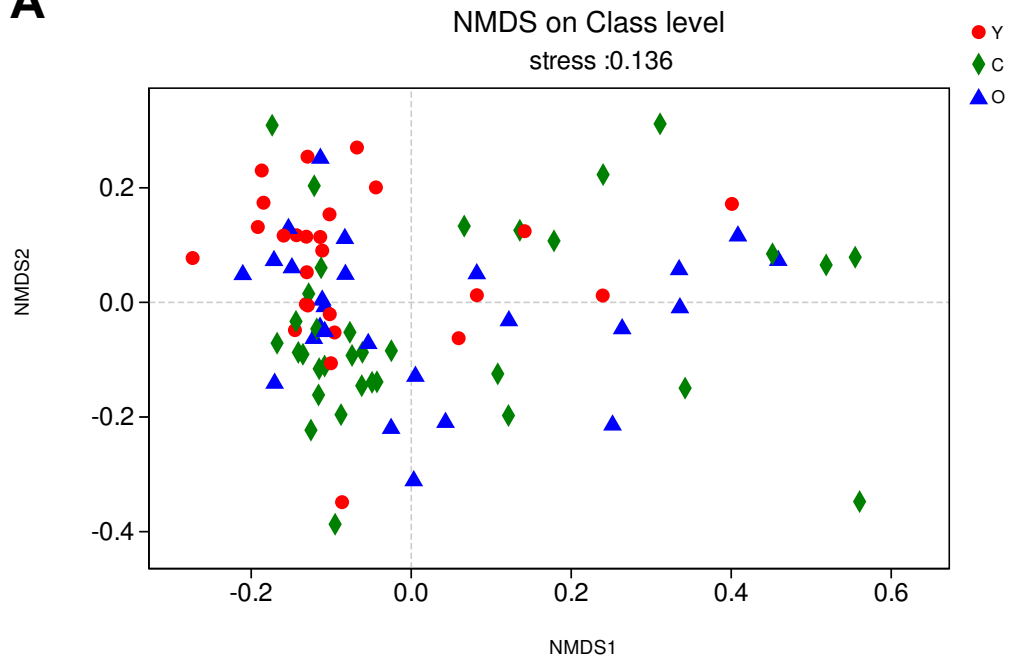

**B**

NMDS on Order level

stress :0.138

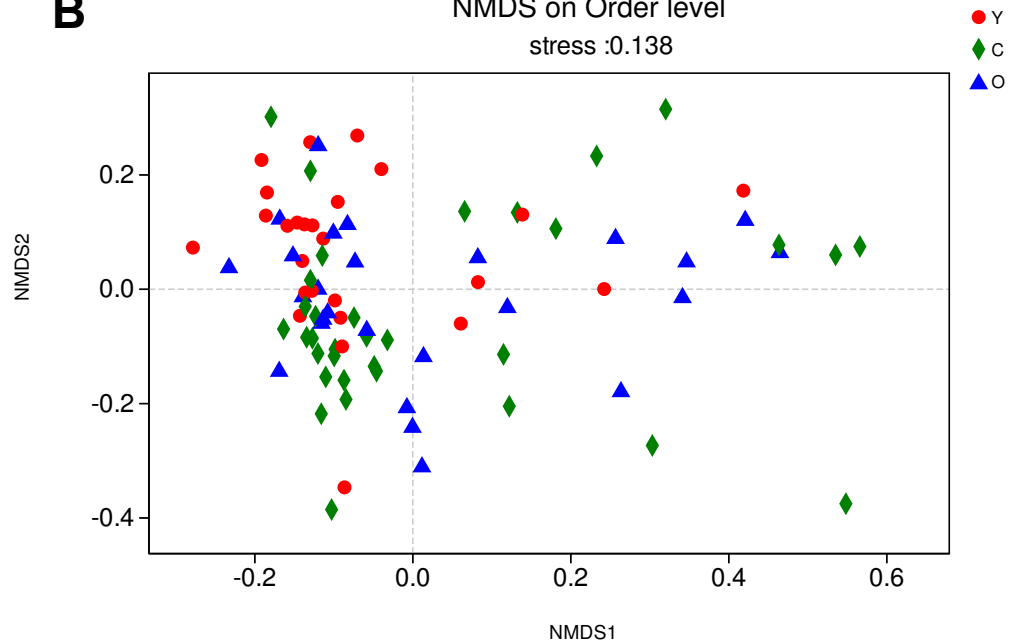

**C**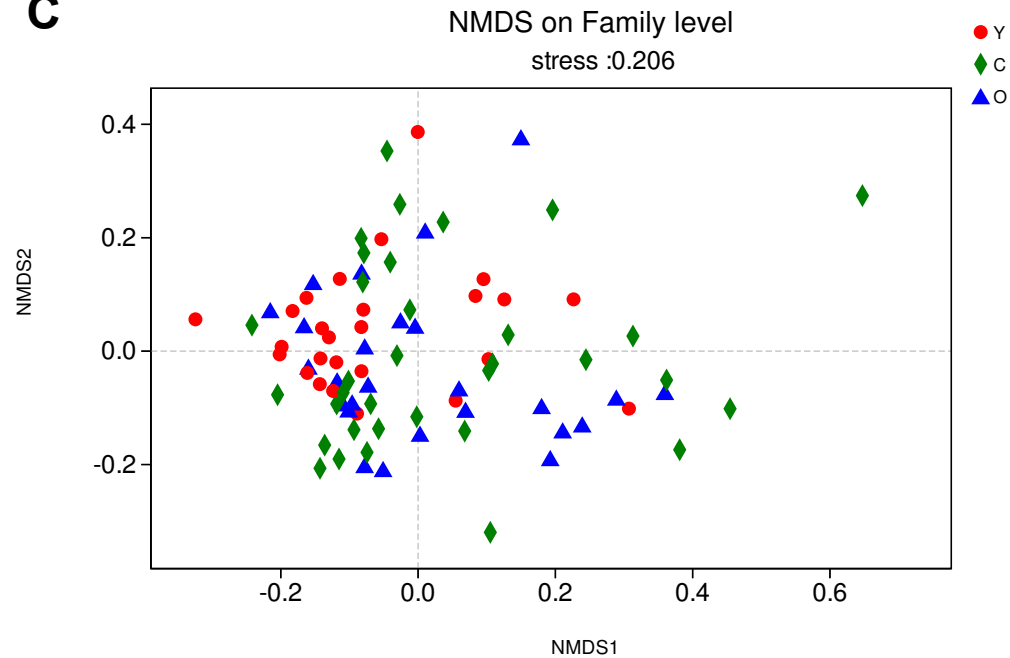

**D**

NMDS on Genus level

stress :0.212

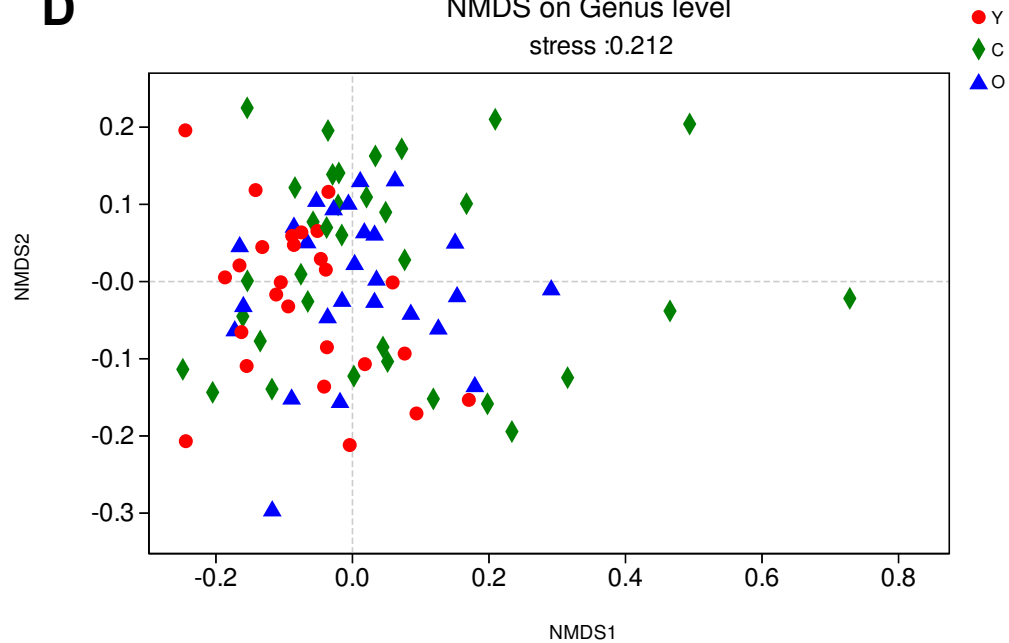

Supplement: Supplementary Figure 3 — (A–D) The results of NMDS on the level of Class, Order, Family, and Genus. (Y: young volunteers; O: old volunteers; C: CRC patients). [file DataSheet_3.pdf]

A

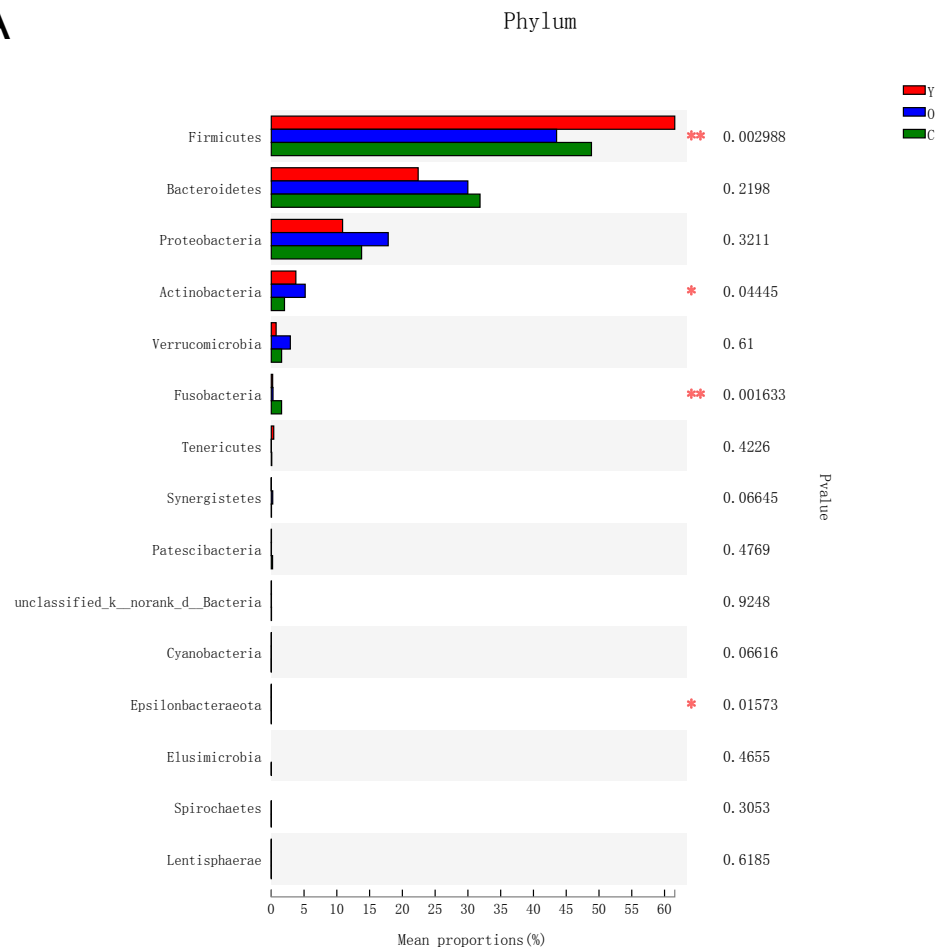

B

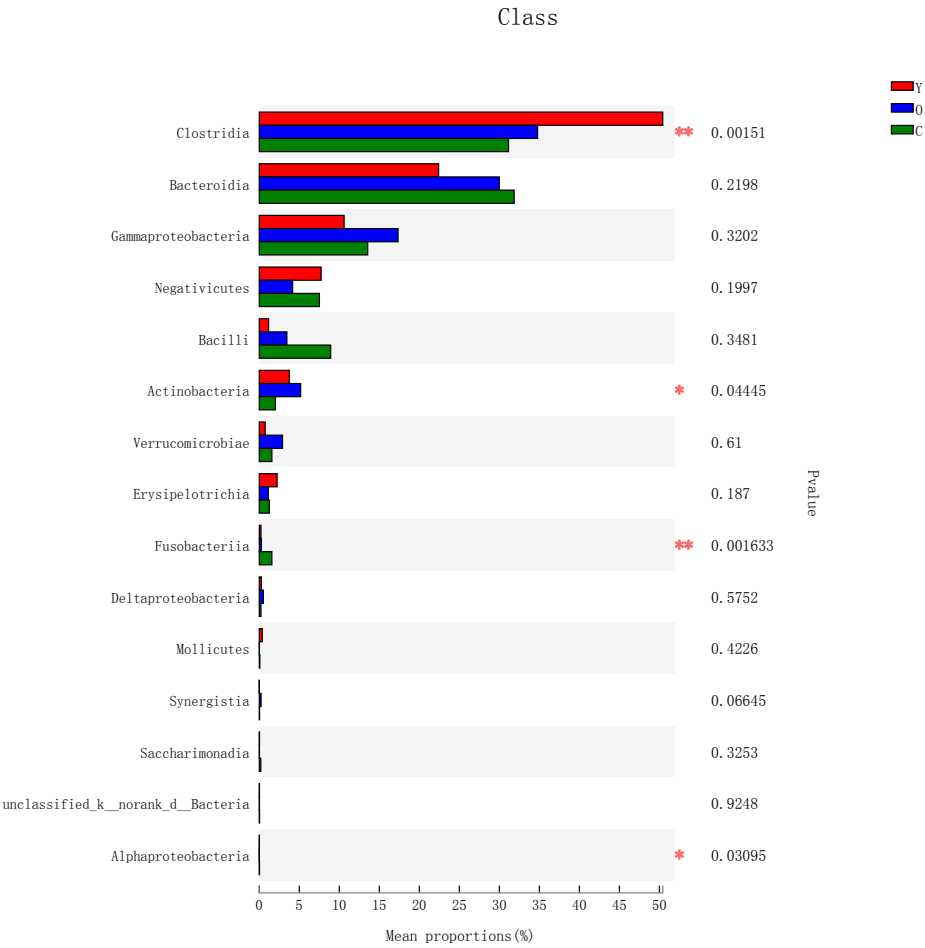

C

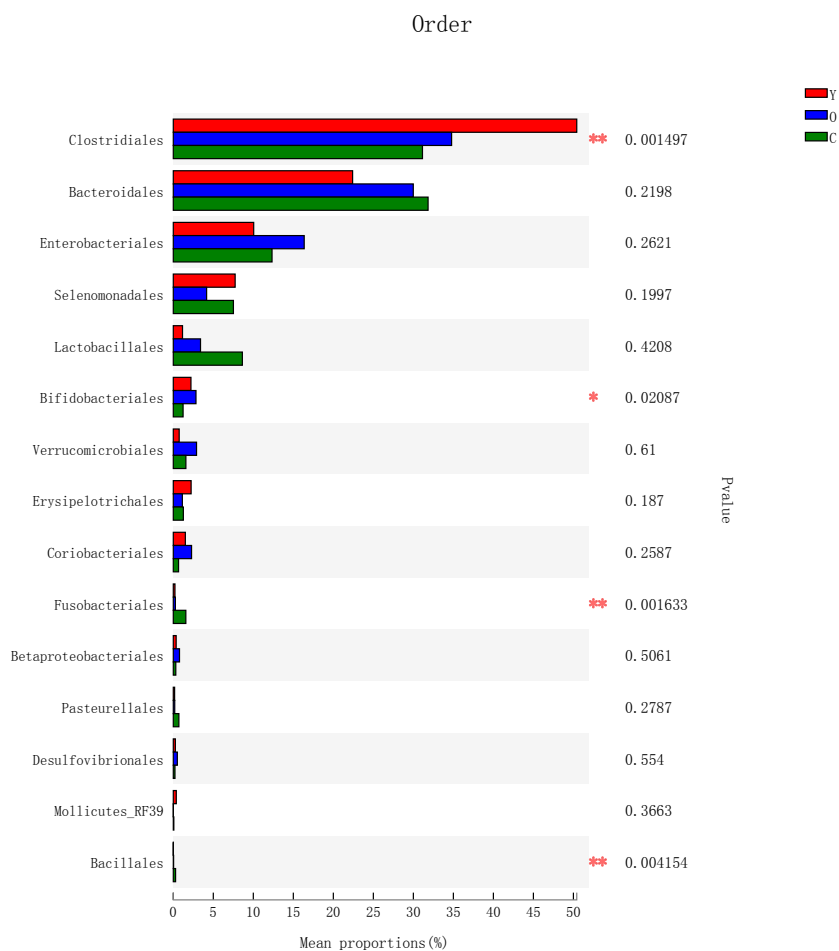

D

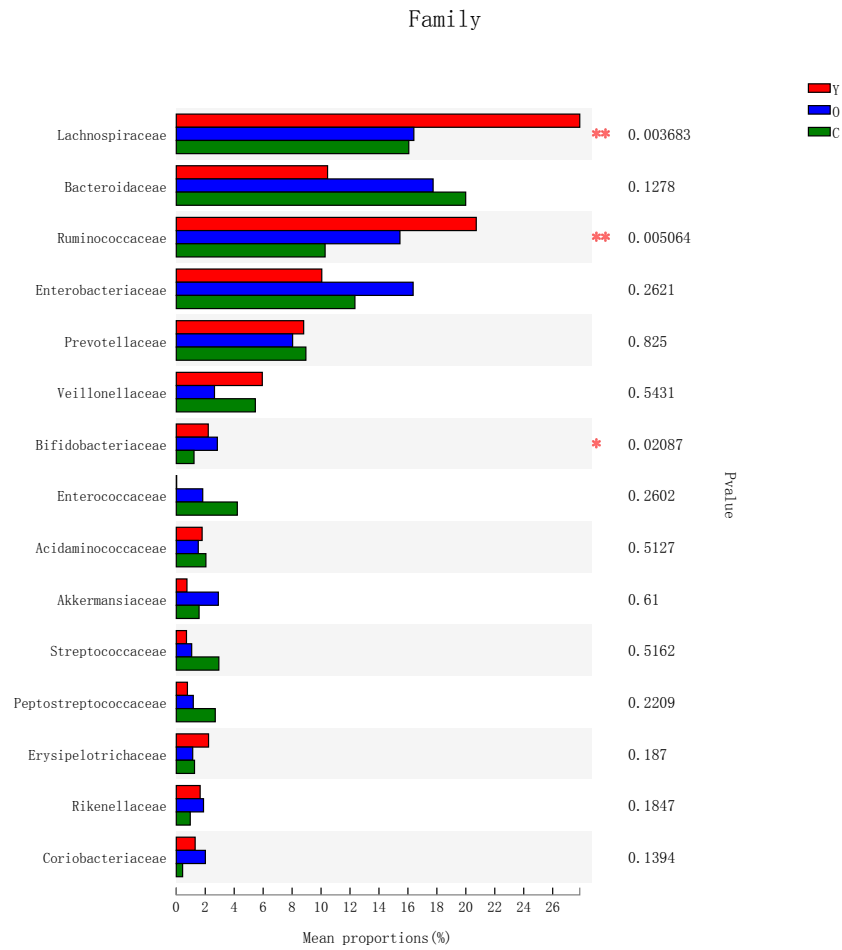

E

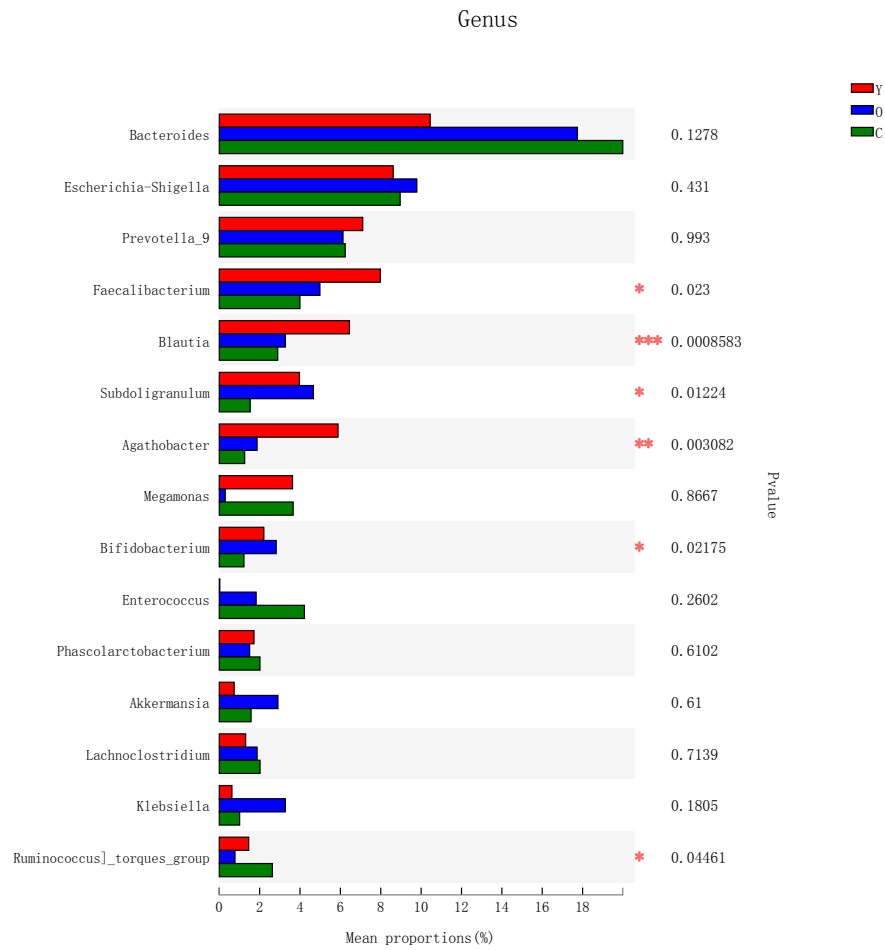

Supplement: Supplementary Figure 4 — (A–E) Distinct distribution of gut bacterial taxa among the three groups on the level of Phylum, Class, Order, Family, and Genus. (Y: young volunteers; O:old volunteers; C: the CRC patients). [file DataSheet_4.pdf]
